# Assessment of Scapulothoracic, Glenohumeral, and Elbow Motion in Adhesive Capsulitis by Means of Inertial Sensor Technology: A Within-Session, Intra-Operator and Inter-Operator Reliability and Agreement Study

**DOI:** 10.3390/s20030876

**Published:** 2020-02-06

**Authors:** Liesbet De Baets, Stefanie Vanbrabant, Carl Dierickx, Rob van der Straaten, Annick Timmermans

**Affiliations:** 1 REVAL Rehabilitation Research, Hasselt University, 3590 Diepenbeek, Belgium; 2 Rehabilitation Sciences and Physiotherapy, Faculty of Rehabilitation Sciences, Hasselt University, 3590 Diepenbeek, Belgium; 3 Department of Physical Medicine and Rehabilitation, Jessa Hospital, 3500 Hasselt, Belgium; 4 Medicine, Faculty of Medicine and Life Sciences, Hasselt University, 3590 Diepenbeek, Belgium

**Keywords:** scapula, frozen shoulder, adhesive capsulitis, reliability, shoulder, kinematic

## Abstract

Adhesive capsulitis (AC) is a glenohumeral (GH) joint condition, characterized by decreased GH joint range of motion (ROM) and compensatory ROM in the elbow and scapulothoracic (ST) joint. To evaluate AC progression in clinical settings, objective movement analysis by available systems would be valuable. This study aimed to assess within-session and intra- and inter-operator reliability/agreement of such a motion capture system. The MVN-Awinda® system from Xsens Technologies (Enschede, The Netherlands) was used to assess ST, GH, and elbow ROM during four tasks (GH external rotation, combing hair, grasping a seatbelt, placing a cup on a shelf) in 10 AC patients (mean age = 54 (±6), 7 females), on two test occasions (accompanied by different operators on second occasion). Standard error of measurements (SEMs) were below 1.5° for ST pro-retraction and 4.6° for GH in-external rotation during GH external rotation; below 6.6° for ST tilt, 6.4° for GH flexion-extension, 7.1° for elbow flexion-extension during combing hair; below 4.4° for GH ab-adduction, 13° for GH in-external rotation, 6.8° for elbow flexion-extension during grasping the seatbelt; below 11° for all ST and GH joint rotations during placing a cup on a shelf. Therefore, to evaluate AC progression, inertial sensors systems can be applied during the execution of functional tasks.

## 1. Introduction

Adhesive capsulitis (AC), or frozen shoulder, is a pathological glenohumeral (GH) joint condition, characterized by adhesions across the GH joint capsule and surrounding ligaments, which negatively affect active and passive GH mobility [1,2]. Movement restrictions generally occur in all movement planes, with more pain towards the end of the available joint motion and with more external rotation restrictions in elevated arm positions [3]. Therefore, AC highly interferes with the independent performance of activities of daily living [3]. A primary, idiopathic form of AC and a secondary form, following trauma or surgery, are described [4]. The incidence of AC is 3% to 5% in the general population [1], with 70% of persons with AC being women [3]. Apart from pain and GH mobility deficits [5,6], AC leads to shoulder dysfunctions and reduces daily life autonomy and quality of life [2]. Although AC is a self-limiting condition, it takes between one to three years to resolve [7,8]. However, mild symptoms may persist for several years in small groups of persons with AC [8]. The treatment goals of medical and physiotherapy treatment in persons with AC are to increase glenohumeral mobility and to normalize scapulothoracic (ST) (mal)adaptive movement patterns, to increase shoulder function [3]. Therefore, the objective assessment of GH motion and compensatory motion in the adjacent scapulothoracic (ST) and elbow joint is of interest to evaluate AC treatment effects and follow up progress [5,6].

The evaluation of active and passive shoulder movement in current clinical practice of AC patients is generally done by visual observation or goniometry [3,9]. Despite the easy-to-use character of these measurements, they have the disadvantage of not being able to measure isolated GH and scapulothoracic motion. Instead, they generally measure humerothoracic motion during active movement assessment. Furthermore, goniometry measurements are typically performed during uniplanar movement (e.g., arm elevation in the frontal plane), instead of during the performance of functional tasks resembling activities of daily life. However, in the last decade, the development of inertial sensor technologies for objective movement assessment of joint range of motion in clinical practice has emerged given their opportunity to measure range of motion during more complex, functional and multiplanar movements [10,11]. Inertial sensor systems are furthermore relatively inexpensive and do not require specific expertise to operate. Some of these systems are currently commercially available and provide, apart from the hardware (inertial motion sensors), software which is necessary to assess motion (i.e., calculate joint angles from the recorded signals of the inertial sensors). As such, these systems seem to be promising tools to use in clinical orthopedic and physiotherapy practice to investigate progression of GH motion in AC patients, and to investigate compensatory patterns in the elbow or ST joint during relevant functional activities.

However, before such a system is implemented in clinical settings, the assessment of the system’s reliability and agreement in calculating joint angles within and between assessors/clinicians should be assessed (i.e., the so-called within-session, intra-operator, and inter-operator reliability and agreement). Knowledge of a system’s measurement error makes interpretation of recorded motion data straightforward (i.e., measurement error can be distinguished from true differences/recovery) [12]. A recent systematic review indicated that the main body of literature on the measurement properties of inertial sensors for the assessment of joint range of motion focused towards the assessment of lower limb joint angles during walking/running [13]. Regarding shoulder complex joint angles, only three studies are currently available, which report appropriate reliability and agreement results for ST kinematic assessment by means of inertial sensors [14,15,16]. However, they only describe the reliability and agreement of ST joint angles during analytical arm elevation tasks. Furthermore, with regard to the reliability of GH joint angles, no literature is available. Instead, two studies were found describing appropriate reliability results for humerothoracic range of movement assessment in persons without shoulder complaints [17,18]. However, in one of these studies, range of motion was assessed during passive arm movement, which does not resemble daily life movement [18].

Apart from the assessment of GH and ST range of motion during analytical arm movement tasks, the assessment of GH and ST range of motion during functional movement tasks (i.e., tasks resembling the activities which are difficult to perform by persons with shoulder complaints), is essential in the evaluation of AC progression and in the evaluation of the effect of AC treatment [3]. Given the lack on reliability and agreement data of GH and ST range of motion assessment by inertial sensors during functional movement tasks [13], and given that AC patients have difficulties performing GH elevation and external rotation due to GH capsular restrictions [3], the aim of this study was to assess within-session, intra-operator, and inter-operator reliability and agreement of a functional movement protocol with a special focus towards GH elevation and external rotation movement tasks, to assess GH, ST, and elbow motion in AC patients. We furthermore aim to formulate recommendations regarding parameter selection when using inertial sensor movement analysis to evaluate treatment efficacy and follow up progress. It is hypothesized that, in line with previous research [14,15,16,17,18], in general good reliability and agreement results will be found for ST, GH, and elbow joint range of motion assessment, but that reliability and agreement results will depend on the complexity of the assessment movement task.

## 2. Materials and Methods

### 2.1. Participants

Ten individuals with primary AC were recruited via the orthopedic department and rehabilitation ward of Jessa Hospital Hasselt (Hasselt, Belgium). Participants were included if they received a diagnosis of adhesive capsulitis by a medical doctor in the past six months before inclusion. This diagnosis was based on the criteria described by The American Physical Therapy Association [3]: (a) 50% loss of passive GH external rotation motion, as compared to the unaffected side; (b) GH motion losses greater than 25% in at least two other GH movements than external rotation, as compared to the unaffected side; (c) pain accompanying motion losses, which is present for at least one month at the time of diagnosis; and (d) the pain and mobility deficits which are described for at least one month at time of diagnosis, have to be stable or worsen during that month. Persons with a bilateral frozen shoulder, a systemic and/or neurologic disease or a self-reported pathologic condition of the cervical/thoracic region, elbow, or wrist/hand were excluded. All participants gave informed consent prior to study participation, as approved by the Ethical Committee of the Jessa Hospital, Belgium (B243201629465).

### 2.2. Data Collection

#### 2.2.1. Instrumentation

**Figure 1:**
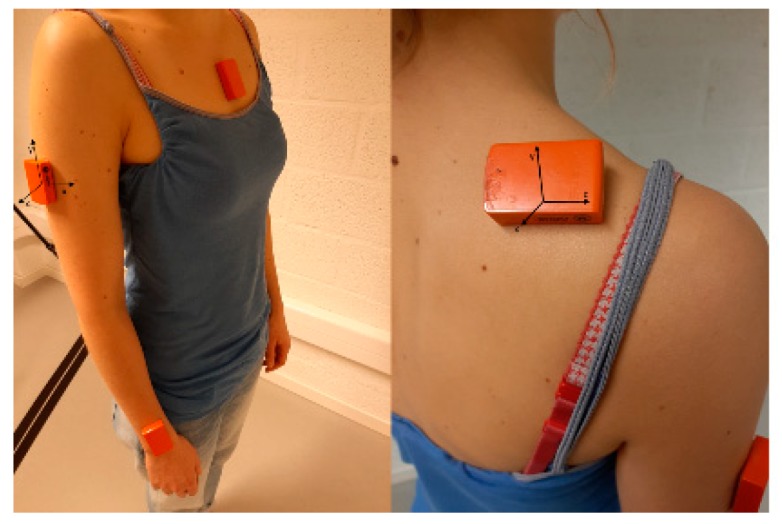
Sensor location and sensor coordinate system.

Kinematic data from the GH, ST, and elbow joint were collected from the affected arm of each person with AC using the commercially available inertial sensor system ‘MVN Awinda® motion capture system’ (Xsens Technologies, Enschede, The Netherlands). Data collection was done via wireless inertial motion sensors (sampling at 60 Hz), consisting of a tri-axial accelerometer, gyroscope, and magnetometer. The signals of the accelerometer, gyroscope, and magnetometer provided the orientation of the technical coordinate system of the sensor relative to a global, earth-based coordinate system. Although only interested in movement data from the affected upper extremity, the system’s upper limb configuration without hands was chosen. Within this configuration, sensors must be placed in the middle of the forehead, on the pelvis, on the sternum and on both scapulae, upper arms, and lower arms. Only the data collected by the sensors on the sternum, and on the scapula, humerus, and lower arm of the affected arm were used for this study. The sternal sensor was positioned on the flat central part of the sternum, the scapular sensor halfway between the scapular trigonum and the acromial angle, in alignment with the upper edge of the scapular spine [19], and the humeral sensor at the central third of the humerus, slightly posterior, at the level of the deltoid insertion. The sensor on the lower arm was positioned on the dorsal side, just proximal of the line between the radial and ulnar styloid process. The position of the sensors is visualized in Figure 1. A static calibration, while standing in upright position, with the upper and lower arm in neutral rotation and the humerus perpendicular to the ground, was performed for the sensor-to-segment calibration [19].

#### 2.2.2. Motion Analysis Protocol for AC

**Figure 2:**
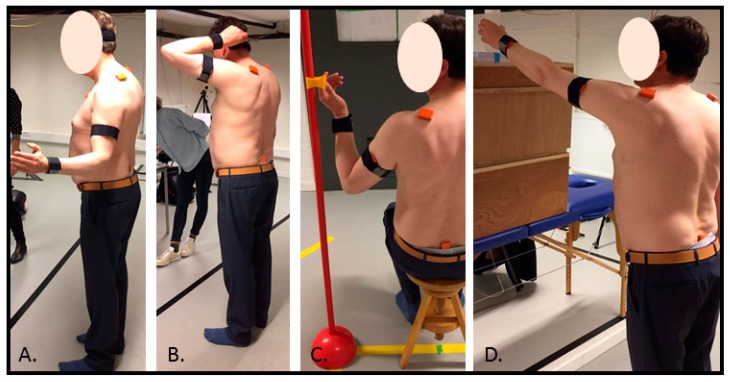
Motion analysis protocol. (**A**) Analytical GH external rotation; (**B**) combing hair; (**C**) grasping a seatbelt; (**D**) placing a cup on an overhead shelf. Remark: in (**C**) the seatbelt is removed to visualize the marker at eye level.

After the calibration, a motion analysis protocol was performed which included the movement tasks which are typically described as difficult to perform by AC patients. The motion analysis protocol included one analytical GH external rotation task, two functional external rotation tasks (grasping the seatbelt and combing hair), and one functional forward flexion task (placing a cup on an overhead shelf) (Figure 2). Details on the protocol’s tasks are outlined in Table 1. Functional tasks were included since functional exercises are, from a motor learning point of view, essential to include in the rehabilitation protocol of AC patients [20]. The focus towards external rotation tasks was based on the fact that GH external rotation loss is the most apparent sign of AC pathology [21]. Participants were instructed to perform the different tasks at a self-selected speed. Each task was first demonstrated and afterwards each participant was given practice trials until the participant was familiar with correct task execution. After these practice trials, five consecutive repetitions per task were recorded.

#### 2.2.3. Data Collection Procedure

Each participant performed three measurement sessions and in each of these sessions the participant performed the motion analysis protocol. One measurement session was performed on the first test occasion and two sessions were performed on the second test occasion. Both test occasions were 2 to 5 days apart, and participants were instructed not to have any medical treatment or perform any physiotherapy/upper extremity exercises between both test occasions. On the first test occasion, the measurement session was accompanied by operator A. On the second test occasion, one measurement session was accompanied by operator A, and the other one by operator B (in randomized order in order to eliminate a potential effect of repeated assessment on the same day). At least 30 min of rest were foreseen between both sessions on the second test occasion. The specific role of the operators per measurement session was (1) the application of the inertial sensors to the upper extremity, (2) the performance of the static calibration trial, and (3) explanation of the movement tasks to the participant.

### 2.3. Data Analysis

MVN Studio software, the software included in MVN-Awinda® motion capture system (Xsens Technologies, Enschede, The Netherlands) was used for motion data analysis. For each anatomical model, and generally based on International Society of Biomechanics recommendations [22], segmental coordinate systems were defined, and scapulothoracic and elbow joint angles were calculated following the ZXY sequence of Euler angles (respective rotations in the sagittal, frontal, and transversal planes). Glenohumeral joint angles were calculated following the XZY Euler sequence. Scapulothoracic kinematics were described in the following three movements: lateral/medial rotation (X), protraction/retraction (Y), and posterior/anterior tilting (Z). Glenohumeral kinematics were reported following abduction/adduction (X), internal/external rotation (Y), and forward flexion/extension (Z). Elbow kinematics were described in one dimension: flexion/extension (Z) (Figure 1).

The first of the five recorded repetitions per session was not selected for data analysis as it could be corrupted by initiation strategies. Movement cycles were defined from start to point of task achievement and checked for erroneous data due to technical errors. Joint angles from all ST, GH, and elbow movements were calculated when the upper extremity was in the start position and at the point of task achievement. Range of motion (ROM), defined as the range of motion between movement start and point of task achievement, was calculated for each ST, GH, and elbow motion for combing hair, grasping a seatbelt, and placing a cup on an overhead shelf. For the analytical GH external rotation task, only ST pro-retraction ROM and GH in-external rotation ROM were calculated.

### 2.4. Statistical Analysis with Regard to Within-Session, Intra-Operator and Inter-Operator Reliability, and Agreement Assessment

Statistical analysis was done using SPSS version 22 (Chicago, IL, USA). Kolmogorov–Smirnov tests confirmed data normality. Means and standard deviations were calculated for each ROM.

Reliability of ROM was calculated using the intraclass correlation coefficient (ICC) plus a 95% confidence interval (95% CI). Agreement was assessed using the standard error of measurement (SEM) and the minimal detectable change (MDC) between two measurement occasions. Furthermore, a proportional SEM (%SEM) and proportional MDC (%MDC) were calculated for proper data interpretation, by expressing SEM and MDC relative to the mean. The following calculations were used: SEM was based on the square root of the mean square error term from the two-way ANOVA [11], MDC was defined as SEM 1.96*$\sqrt{2}$. %SEM and %MCD were described as (SEM/mean)*100 and (MCD/mean)*100, respectively.

Single data (i.e., the data from each analyzed repetition (*n* = 4)) from the first session was used to calculate within-session reliability/agreement (ICCw (2,1); within-session standard error of the measurement (SEMw)). Averaged data of the four repetitions per session completed by the same operator on different test occasions was used for intra-operator reliability/agreement assessment (ICCintra-operator (2,k); SEMintra-operator; MDCintra-operator). From each of the two sessions on the second test occasion (each accompanied by a different operator), averaged data of the four repetitions per task was used for inter-operator reliability/agreement assessment (ICCinter-operator (2,k); SEMinter-operator; MDCinter-operator) [15,16,17]. ICCs > 0.80 were considered substantial, 0.61–0.80 moderate, 0.41–0.60 fair, 0.11–0.40 slight, and 0–0.10 virtually no reliability [23].

For intra-operator and inter-operator agreement assessment, Bland–Altman plots were constructed to graphically display the data of an individual person’s differences between test sessions relative to the respective individual mean, and to examine the data distribution around the zero line. The 95% limits of agreement (LOAs) were calculated (mean difference ± 1.96* SDmean difference) to identify systematic variance (i.e., zero line not included in the 95% CI) or outliers.

## 3. Results

### 3.1. Participants

Ten participants with AC (mean age (SD) = 54 (±6), 7 females) were included. Mean time since diagnosis (SD) was 12 (± 5) weeks.

### 3.2. Reliability and Agreement

Means (SD), SEMs, and %SEMs for within-session agreement assessment are reported in Table 2. Intra-operator and inter-operator means (SD) and agreement outcomes (SEMs, MDCs, %SEMs, %MDCs, 95% LOA) are described in Table 3. Bland–Altman plots for intra-operator and inter-operator agreement assessment are provided in Supplementary Materials.

Within-session, intra-operator, and inter-operator reliability results are reported for each task and assessed ROM in Table 4 and Table 5.

#### 3.2.1. Analytical Glenohumeral External Rotation

SEMsw/%SEMsw for ST pro-retraction and GH in-external rotation were 0.4°/17.8% and 2.3°/15.5%, respectively. Intra-operator and inter-operator SEM/%SEM were higher (range 1°–4.6°/range 11.7%–49.2%), with SEMsinter-operator being higher than SEMsintra-operator.

ICCsw and ICCsintra-operator for ST pro-retraction and GH in-external rotation, and ICCinter-operator for ST pro-retraction showed substantial reliability, while ICCinter-operator for GH in-external rotation showed slight reliability.

#### 3.2.2. Combing Hair

Lowest SEMsw were found for all ST, GH, and elbow ROMs, with %SEMsw of ST ROMs being slightly higher than %SEMsw of GH and elbow ROMs. For (%)SEMintra-operator and (%)SEMinter-operator, the highest values were generally found for ST pro-retraction and GH in-external rotation (%SEM range 30.1%–68.1%). (%)SEMintra-operator and (%)SEMinter-operator for the other ST and GH ROMs and for elbow flexion-extension ROM were lower (%SEM range 4.9%–30.4%).

#### 3.2.3. Grasping the Seatbelt

Highest (%)SEMsw were found for ST pro-retraction and GH flexion-extension, with reported %SEMsw of 43.7% and 23.3%, respectively. For (%)SEMintra-operator and (%)SEMinter-operator, ST pro-retraction and GH flexion-extension showed the highest values (i.e., %SEMs ranged between 37.9 and 71.7). Lowest (%)SEMsw, (%)SEMintra-operator, and (%)SEMinter-operator were described for GH ab-adduction, GH in-external rotation, and elbow flexion-extension (%SEMs range 5.5% and 22.4%).

All ICCsw, ICCsintra-operator, and ICCsinter-operator showed substantial reliability, with the exception of the ICCsinter-operator for ST medial-lateral rotation (0.24), GH ab-adduction (0.74), GH flexion-extension (0.74), and the ICCintra-operator of GH ab-adduction (0.73).

#### 3.2.4. Placing a Cup on an Overhead Shelf

Highest (%)SEMsw were found for ST pro-retraction and elbow flexion-extension, with reported %SEMsw of 21.7% and 30.6%, respectively. With regard to (%)SEMintra-operator and (%)SEMinter-operator, the highest values were reported for elbow flexion-extension (86.2% and 131.3%, respectively). For the ST and GH ROMs, %SEMsintra-operator and %SEMsinter-operator were between 9.5% and 31.2%.

All ICCsw, ICCsintra-operator, and ICCsinter-operator showed substantial reliability, with the exception of the ICCsinter-operator for ST tilting (0.78), and the ICCintra-operator of GH flexion-extension (0.68) and elbow flexion-extension (0.40).

For all tasks, ICCs lower than 0.80 (less than substantial reliability) had moreover systematically large confidence intervals. In addition, large confidence intervals were observed for ST pro-retraction during the grasping the seatbelt task and the combing hair task.

## 4. Discussion

Nowadays, commercially available inertial measurement systems for human movement analysis are emerging. This study assesses the reliability and agreement of ST, GH, and elbow joint ROM assessment in AC patients using a commercially available inertial measurement system. Such a system is of great value to evaluate treatment effects in persons with AC in daily practice. However, before such a system can be clinically implemented, the establishment of its reliability and knowledge of its measurement errors in the assessment of ST, GH, and elbow joint ROM is essential.

The reliability and agreement results, reported in this study, were not sufficient overall, indicating that that the clinical implementation of inertial sensor technology in the assessment of shoulder movement in persons with AC is not straightforward at this moment. The values of the SEMs/MDCs and of the ICCs (together with the width of their confidence intervals) were dependent on the assessed joint rotation and assessed task. Furthermore, task-specific differences in within-session, intra- and inter-operator reliability, and agreement were found.

However, results were comparable to reported reliability and agreement results of other studies assessing the reliability and agreement of ST or humerothoracic range of motion measurements by means of inertial sensors in healthy persons. Schiefer et al. [18] reported a within-session ICC for humerothoracic abd-adduction of 0.96 in healthy persons which is in line with the reported within-session ICC-values in this study during functional task performance in persons with AC [18]. Bouvier et al. [17] described the performance of standardized analytical movements in healthy persons; there were lower intra-operator reliability results for humerothoracic abd-adduction and ext-internal rotation than for flexion-extension [17]. This was not consistent with the results reported in this study, since a better reliability in GH flexion-extension than in GH abd-adduction or GH ext-internal during the performance of functional tasks was not consistently observed. Within-session agreement results (SEMs) for ST rotations, reported in healthy persons by Parel et al. [15] (i.e., between 1.2°–3.9°, 1.8°–3.4°, and 1.4°–2.8° for scapulothoracic pro-retraction, med-lateral rotation and antr-posterior tilt) were generally higher than the reported within-session SEMs in this study [15]. Furthermore, van den Noort et al. [16] reported intra- and inter-operator SEMs for ST med-lateral rotation and ant-posterior tilt lower than 5°, which is generally in line with the reported intra- and inter-operator SEMs in this study [16]. Reported intra- and inter-operator ICCs by van den Noort et al. [16] during analytical arm elevation movements (pro-retraction ICC range 0.65–0.85 and med-lateral rotation ICC range 0.56–0.91) were slightly lower than the reported ICC in this study, indicating better slightly better reliability during functional task performance [16].

In order to fully investigate the reliability and agreement of the inertial sensor system in the assessment of joint ROM in AC patients, within-session reliability and agreement was assessed together with intra- and inter-operator reliability and agreement. While within-session data provides information on reliability and measurement error caused by intra-subject movement variability (natural source of variability), intra-operator (between-session) and inter-operator reliability and agreement provide information on additional sources of error (e.g., task-standardization/explanation; manual handling during sensor placement/calibration). Specifically regarding the applied inertial sensor technology used in this study, following sources of errors must be considered: calibration inaccuracies when positioning the participant in the neutral calibration position; palpation inaccuracies when placing the sensors on the segments in alignment with the segment orientation; measurement errors when determining anthropometric dimensions which serve as necessary input for the upper body configuration/model; and inaccuracies in task-explanation. The fact that both intra- and inter-operator reliability and agreement are assessed, additionally provides information about the source of error which is seen between sessions: if the error is operator-dependent (e.g., personal manner of task explanation or placing sensors), better intra-operator than inter-operator reliability and agreement are expected. When intra-operator and inter-operator reliability/agreement are equal but worse than within-session data, natural variability in task-execution by testing on different occasions might be assumed.

Furthermore, it is known that the magnitude of the ICC is dependent on between-subject variability (i.e., high ICCs can hide poor trial-to-trial consistency in case of high between-subject variability) [12]. Conversely, limited between-subject variability could result in poor ICCs even when trial-to-trial consistency is high. Therefore, ICCs should always be considered in conjunction with agreement parameters (SEMs, MDC, LOA). Since measurement errors have the same unit as the measurement of interest (in this case degrees), they are easily interpretable in clinical practice. Finally, the range of ICCs’ confidence intervals should be taken into account when interpreting the value of the reported ICC.

When interpreting the reported results based on the aforementioned information, recommendations for task selection can be made, in order to make specific guidelines on the appropriate use of inertial sensors systems for range of motion measurements in clinical practice. For the analytical GH external rotation, within-session and intra-operator agreement were similar for GH in-external rotation. In contrast, inter-operator agreement was remarkably lower for GH in-external rotation. In addition, for ST pro-retraction, inter-operator agreement was lower than intra-operator agreement. As this difference between intra- and inter-operator agreement is only notable during the analytical GH external rotation, differences in sensor placement between the operators are not expected. The agreement differences might rather be explained by the fact that the analytical external rotation task was the only task without a clear endpoint, making task standardization by thorough task explanation and clear task instructions crucial. As such, the lower inter-operator agreement results are expected to relate to inconsistent task explanation/instructions.

For the functional external rotation tasks (i.e., combing hair and grasping a seatbelt), different joint ROMs show different reliability and agreement results. For combing hair, better agreement results are reported for ST med-lateral rotation, GH flexion-extension, and elbow flexion-extension, indicating low intra-individual natural movement variability for these motions while combing hair. In contrast, given the lower agreement results for transversal plane motion during combing hair, this task is not recommended to assess ST pro-retraction and/or GH internal-external rotation. It seems a better choice to assess GH in-external rotation during the grasping the seatbelt task. For this task, best agreement results are reported for GH in-external rotation, GH ab-adduction, and elbow flexion-extension. On the other hand, grasping the seatbelt is not the optimal option when assessment of ST motion is of primarily interest, given the worse agreement results for ST medial-lateral rotation, ST tilting, and ST pro-retraction. Placing a cup on an overhead shelf is the task with the best agreement results across all ST and GH joint motions. Only for elbow flexion-extension, worse agreement results are reported. Probably the fact that this task is executed in one movement plane (i.e., the sagittal plane), in contrast to combing hair and grasping the seatbelt which are tasks consisting of movements in all movement planes, adds to the good agreement results for all ST and GH ROMs during placing a cup. Recommendations for parameter selection are summarized in Table 6.

This protocol, consisting of four tasks during which ST, GH, and elbow kinematics are measured by means of a commercially available inertial sensor system, is only reliable and shows agreement within and between operators when specific recommendations for parameters selection (i.e., specific ROMs during specific tasks) are applied. Given that one measurement session only lasts for 15 minutes, this protocol seems usable in daily physiotherapy or orthopedic practice, when relying on these recommendations. However, when inspecting the magnitude of ST ROMs, one might question their validity. When comparing to literature, reported ST ROM seems underestimated for lateral rotation and overestimated for posterior tilting [5,6,16,24,25,26]. Parel et al. [14,15] and van den Noort et al. [26] reported good validity results for inertial sensor-based ST joint motion during arm elevation tasks, when an YZX-sequence for ST joint angle calculation was used [14,15,16,26]. However, the software included in this commercially available package uses the ZXY-sequence for ST joint angle calculation, which might be the cause of over- or underestimation of ST tilting and lateral rotation ROM, respectively. Therefore, it is essential to further clarify these validity concerns on ST joint angle calculation during the proposed protocol before it is clinically applicable.

There are several limitations of this study. Given that the reported movement protocol was specifically designed for AC patients (i.e., persons with shoulder movement restrictions towards arm elevation and external rotation), one could argue that the reported SEM and MDC values are only applicable to persons with these shoulder movement restrictions, which decreases the generalizability of the results into different patient populations. However, the fact that arm abduction and external rotation movement restrictions often occur in other shoulder joint problems than AC, increases the relevance of the developed protocol for persons with other than AC shoulder joint conditions.

Only 10 persons with AC were included in this study which might be a limitation. For between-session reliability assessment, it is essential that all conditions are as similar as possible between the two assessment sessions, to exclude as much ‘noise’ as possible. An example of ‘noise’ is potential improvement in mobility between the two test sessions due to, for example, treatment or the performance of exercises. Given that mobility exercises and physiotherapy are the main treatment in persons with AC, participation in this study interfered with their normal rehabilitation. Therefore, only 10 participants were included.

## 5. Conclusions

This study indicates that commercially available inertial sensor systems have appropriate reliability and agreement for use in daily practice of persons with AC, when recommendations for parameter selection are followed.

## Figures and Tables

**Table 1:** Description of the tasks included in the assessment protocol.

Task	Explanation
Analytical GH external rotation	This task started with the test person in standing position, feet at hip width, and the tested arm in a 90° elbow flexion, with the upper arm alongside the body. The person was asked to externally rotate the humerus in the glenohumeral (GH) joint by moving the lower arm outwards. The person was asked not to move the scapula or the thorax. The end position was held for two seconds before going back to the start position.
Combing hair	This task started with the test person in standing position, feet at hip width, and the arms alongside the body. In the tested hand, the person held a comb. The person was asked to imitate combing hair, starting from the front of the head. The task ended when the comb was on the back of the head, with the hand palm facing the occiput. The person was asked to hold this end position for 2 s and to return afterwards to the start position.
Grasping a seatbelt	This task started with the test person in sitting position on a chair without back support, feet at hip width, knees in a 90° flexion, and hands placed on the thighs. A seatbelt was placed at a standardized distance from the midpoint of the chair (distance between belt and midpoint chair was 110% from the interacromial distance), with a marker at eye level. The person was asked to grab the seatbelt with the ipsilateral thumb and index finger at height of the marker and to hold that position for two second before going back to the start position.
Placing a cup on an overhead shelf	This task started with the test person in standing position, feet at hip width, and arms alongside the body. The person held a cup in the tested hand. The height of the shelf and the distance from the standing position to the shelf was adjusted according to the participant’s height (at 93% of the participant’s height) and arm length (distance acromion to the base of the third metacarpal, distance at 115% of arm length), respectively. The task ended when the cup was placed on a marked spot on the shelf. The participant held the arm for 2 s on the endpoint before coming back to the start position.

**Table 2:** Within-session means, standard deviations, and standard error of measurements, expressed in degrees.

	Analytical Ext Rot			Seatbelt			Comb Hair			Cup		
	Mean (SD)	SEMw	%SEMw	Mean (SD)	SEMw	%SEMw	Mean (SD)	SEMw	%SEMw	Mean (SD)	SEMw	%SEMw
Scapulothoracic												
Med-Lat rotation	/	/	/	−6.7 (5.2)	1.3	19.5	−9.0 (6.9)	1.2	13.0	−9.5 (5.6)	1.0	10.1
Pro-retraction	2.4 (2.7)	0.4	17.8	3.8 (4.1)	1.6	43.7	6.9 (7.3)	1.1	16.1	−9.0 (7.6)	2.0	21.7
Tilting	/	/	/	−15.5 (9.0)	1.2	7.5	−25.7 (5.7)	2.3	9.1	−19.4 (10.4)	1.9	9.8
Glenohumeral												
Abd-adduction	/	/	/	−44.3 (14.1)	2.4	5.5	−69.3 (29.8)	4.7	6.8	−50.7 (21.6)	4.3	8.4
In-external rotation	15.1 (6.0)	2.3	15.5	60.6 (20.5)	3.9	6.4	54.0 (27.0)	3.7	6.8	34.2 (23.4)	5.5	16.1
Flexion-extension	/	/	/	−18.9 (14.0)	4.4	23.3	−54.2 (8.8)	2.9	5.4	−55.8 (10.9)	1.0	1.8
Elbow												
Flexion-extension	/	/	/	−50.0 (17.2)	4.5	9.0	−143.8 (11.0)	2.7	1.9	−5.4 (4.5)	1.6	30.6

SEMw = within-session standard error of the measurement; %SEM_w_ = (SEM_w_/mean); analytical ext rot = analytical glenohumeral external rotation; seatbelt = grasping the seatbelt; comb hair = combing hair; cup = placing a cup on an overhead shelf. Med-lat rotation = medial-lateral rotation; pro-retraction = protraction-retraction; tilting = anterior-posterior tilting; Abd-adduction = abduction-adduction; in-external rotation = internal-external rotation. Positive value when movement toward scapulothoracic medial rotation, retraction, anterior tilt; glenohumeral adduction, external rotation, extension; elbow extension.

**Table 3:** Intra-operator and inter-operator means, standard deviations, standard error of measurements, minimal detectable changes and 95% limits of agreement expressed in degrees, for the different tasks.

	Intra-Operator Agreement								Inter-Operator Agreement							
	Mean (SD)	SEM	%SEM	MDC	%MDC	Mean_diff_	SD_diff_	LOA	Mean (SD)	SEM	%SEM	MDC	%MDC	Mean_diff_	SD_diff_	LOA
**Analytical Glenohumeral External Rotation**																
	Scapulothoracic															
Pro-retraction	2.7 (2.8)	1.0	36.0	2.7	99.7	−0.6	1.2	1.7, −3.0	3.1 (3.0)	1.5	49.2	4.3	136.4	−0.2	1.7	3.3, −3.7
	Glenohumeral															
Int-external rotation	15.7 (6.3)	1.8	11.7	5.1	32.5	−1.1	4.8	8.4, −10.7	16.6 (6.5)	4.6	27.5	12.7	76.3	−0.8	8.4	16.1, −17.7
**Combing Hair**																
	Scapulothoracic															
Med-lat rotation	−9.5 (7.2)	2.9	30.4	8.0	84.4	1.0	3.4	7.9, −5.9	−9.8 (7.0)	0.9	9.6	2.6	26.5	−0.4	1.7	3.0, −3.9
Pro-retraction	5.3 (6.4)	3.6	68.1	10.1	188.8	3.1	4.6	12.2, −6.0	4.4 (5.2)	1.8	40.8	5.0	113.1	−1.2	3.0	4.7, −7.2
Tilting	−25.2 (5.2)	3.6	14.3	10.0	39.5	−1.1	4.1	7.2, −9.4	−24.2 (5.0)	6.6	27.2	18.3	75.5	−0.9	7.1	13.3, −15.1
	Glenohumeral															
Abd-adduction	−71.7 (29.5)	9.5	13.2	26.3	36.7	5.6	12.6	30.8, −19.5	−78.3 (26.3)	19.1	24.5	53.1	67.8	2.3	22.1	46.5, −41.9
Int-external rotation	54.7 (27.4)	16.5	30.1	45.6	83.3	−2.1	19.1	36.1, −40.3	60.4 (27.0)	27.8	46.0	77.0	127.5	−5.6	33.2	60.9, −72.1
Flexion-extension	−53.0 (9.1)	5.0	9.4	13.8	26	−2.3	6.2	10.0, −14.7	−54.6 (10.1)	6.4	11.7	17.6	32.3	5.4	8.0	21.4, −10.6
	Elbow															
Flexion-extension	−144.2 (12.3)	7.1	4.9	19.7	13.6	0.7	7.8	16.3, −14.8	142.2 (15.0)	4.3	11.8	11.8	8.3	−4.7	12.5	20.2, −29.7
**Grasping the Seatbelt**																
	Scapulothoracic															
Med-lat rotation	−6.7 (4.8)	2.0	29.9	5.6	83.0	0.1	3.4	7.0, −6.8	−6.7 (3.9)	3.4	50.6	9.4	140.3	−0.2	4.1	8.1, −8.5
Pro-retraction	3.0 (4.5)	2.2	71.7	6.0	198.6	2.4	2.5	7.5, −2.7	2.8 (5.1)	1.3	44.8	3.5	124.3	−1.0	2.5	4.1, −6.07
Tilting	−14.7 (7.5)	5.1	34.7	14.1	96.1	−1.5	6.1	10.7, −13.8	−15.4 (6.0)	3.8	24.5	10.5	68.0	2.9	4.3	11.5, −5.8
	Glenohumeral															
Abd-adduction	−47.8 (12.0)	3.7	7.6	10.1	21.2	6.8	9.8	26.5, −12.8	−50.8 (9.6)	4.4	8.6	12.1	23.9	−0.9	−0.8	11.8, −13.3
Int-external rotation	61.3 (20.7)	9.9	16.2	27.4	44.8	−1.3	15.0	28.6, −31.4	58.0 (21.1)	13.0	22.4	36.0	62.0	7.9	17.9	43.4, −27.7
Flexion-extension	−17.0 (13.3)	6.4	37.9	17.9	105.0	−3.8	7.4	10.9, −18.6	−19.0 (11.8)	7.8	41.0	21.6	113.6	4.7	9.8	24.3, −14.9
	Elbow															
Flexion-extension	−49.8 (16.3)	4.2	8.4	11.5	23.1	−0.3	11.4	22.5, −23.0	−50.6 (18.3)	6.8	13.4	18.7	37.0	1.8	10.6	23.0, −19.4
**Placing a Cup on an Overhead Shelf**																
	Scapulothoracic															
Med-lat rotation	−9.7 (6.2)	2.3	23.8	6.4	66.1	0.0	3.7	7.6, −7.0	−9.9 (6.1)	3.3	33.7	9.2	93.4	0.2	3.8	7.7, −7.3
Pro-retraction	−10.1 (6.2)	3.1	31.0	8.6	85.9	2.1	3.9	10.0, −5.8	−10.5 (5.0)	1.5	14.4	4.2	39.8	−1.3	2.2	3.1, −5.7
Tilting	−18.6 (9.0)	4.3	22.9	11.8	63.6	−1.6	5.9	10.2, −13.5	−17.4 (7.5)	3.2	18.5	8.9	51.3	−0.7	3.8	6.9, −8.3
	Glenohumeral															
Abd-adduction	−52.3 (20.9)	6.4	12.2	17.7	33.8	3.2	8.9	20.9, −14.6	−53.6 (17.6)	6.1	11.3	16.9	31.4	−3.3	7.5	11.6, −18.3
Int-external rotation	35.4 (21.2)	11.0	31.2	30.6	86.5	−2.4	13.7	25.1, −29.8	36.9 (21.0)	10.2	27.7	28.3	76.7	−0.7	19.1	37.4, −38.8
Flexion-extension	−53.8 (11.2)	9.4	17.6	26.2	48.7	−4.0	11.0	18.0, −26.1	−56.4 (12.9)	5.4	9.5	14.8	26.3	9.2	6.7	22.6, −4.2
	Elbow															
Flexion-extension	−5.0 (4.0)	4.3	86.2	11.8	238.8	−0.3	6.2	12.2, −12.8	−3.7 (4.3)	4.9	131.3	13.6	363.8	−1.5	6.6	11.8, −14.8

SEM = standard error of the measurement; MDC = minimal detectable change; %MDC = (MDC/Mean_b_); %SEM = (SEM_b_/Mean_b_); LOA = Bland–Altman 95% limits of agreement; Mean_Diff_ = mean of the differences between two test sessions. Med-lat rotation = medial-lateral rotation; pro-retraction = protraction-retraction; tilting = anterior-posterior tilting; Abd-adduction = abduction-adduction; in-external rotation = internal-external rotation. Positive value when movement toward scapulothoracic medial rotation, retraction, anterior tilt; glenohumeral adduction, external rotation, extension; elbow extension.

**Table 4:** Intraclass correlation coefficients for within-session reliability.

	Analytical Ext Rot	Seatbelt	Comb Hair	Cup
ICCw (95% CI)				
Scapulothoracic				
Med-Lat rotation	/	0.97 (0.92, 0.99)	0.98 (0.94, 0.99)	0.97 (0.93, 0.99)
Pro-Retraction	0.89 (0.74, 0.97)	0.92 (0.90, 0.98)	0.97 (0.94, 0.99)	0.96 (0.89, 0.99)
Tilting	/	0.98 (0.95, 0.99)	0.87 (0.72, 0.96)	0.97 (0.93, 0.99)
Glenohumeral				
Abd-adduction	/	0.98 (0.95, 0.99)	0.98 (0.96, 0.99)	0.97 (0.91, 0.99)
In-external rotation	0.85 (0.65, 0.96)	0.98 (0.95, 0.99)	0.98 (0.96, 0.99)	0.96 (0.89, 0.99)
Flexion-extension	/	0.96 (0.91, 0.99)	0.88 (0.71, 0.97)	0.97 (0.91, 0.99)
Elbow				
Flexion-extension	/	0.97 (0.92, 0.99)	0.94 (0.85, 0.98)	0.90 (0.76, 0.97)

95% CI = 95% confidence interval; ICCw = intraclass correlation coefficient within session; analytical ext rot = analytical glenohumeral external rotation; seatbelt = grasping the seatbelt; comb hair = combing hair; cup = placing a cup on an overhead shelf. Med-lat rotation = medial-lateral rotation; pro-retraction = protraction-retraction; tilting = anterior-posterior tilting; Abd-adduction = abduction-adduction; in-external rotation = internal-external rotation.

**Table 5:** Intraclass correlation coefficients for intra- and inter-operator reliability.

	Analytical Ext Rot	Seatbelt	Comb Hair	Cup
ICCintra-operator (95% CI)				
Scapulothoracic				
Med-Lat rotation	/	0.87 (0.46, 0.97)	0.94 (0.78, 0.99)	0.92 (0.66, 0.98)
Pro-Retraction	0.95 (0.78, 0.99)	0.82 (0.04, 0.93)	0.81 (0.23, 0.95)	0.87 (0.51, 0.97)
Tilting	/	0.82 (0.29, 0.96)	0.82 (0.31, 0.96)	0.89 (0.56, 0.97)
Glenohumeral				
Abd-adduction	/	0.73 (0.02, 0.93)	0.95 (0.83, 0.99)	0.95 (0.83, 0.99)
In-external rotation	0.85 (0.35, 0.97)	0.87 (0.46, 0.97)	0.89 (0.50, 0.96)	0.90 (0.59, 0.97)
Flexion-extension	/	0.91 (0.63, 0.98)	0.87 (0.51, 0.97)	0.68 (0.00, 0.92)
Elbow				
Flexion-extension	/	0.88 (0.50, 0.97)	0.90 (0.60, 0.98)	0.40 (0.00, 0.85)
ICCinter-operator (95% CI)				
Scapulothoracic				
Med-Lat rotation	/	0.24 (0.00, 0.82)	0.94 (0.75, 0.98)	0.82 (0.23, 0.96)
Pro-Retraction	0.83 (0.45, 0.96)	0.91 (0.64, 0.98)	0.83 (0.39, 0.96)	0.86 (0.45, 0.97)
Tilting	/	0.87 (0.50, 0.97)	0.29 (0.00, 0.83)	0.78 (0.18, 0.95)
Glenohumeral				
Abd-adduction	/	0.74 (0.05, 0.88)	0.80 (0.23, 0.96)	0.93 (0.51, 0.97)
Int-external rotation	0.38 (0.00, 0.87)	0.84 (0.42, 0.96)	0.73 (0.00, 0.94)	0.87 (0.49, 0.97)
Flexion-extension	/	0.74 (0.00, 0.95)	0.83 (0.37, 0.96)	0.84 (0.35, 0.96)
Elbow				
Flexion-extension	/	0.85 (0.38, 0.96)	0.79 (0.22, 0.95)	0.84 (0.35, 0.96)

95% CI = 95% confidence interval; ICCintra-operator = intraclass correlation coefficient within one operator; ICCinter-operator = intraclass correlation coefficient between two operators; analytical ext rot = analytical glenohumeral external rotation; seatbelt = grasping the seatbelt; comb hair = combing hair; cup = placing a cup on an overhead shelf. Med-lat rotation = medial-lateral rotation; pro-retraction = protraction-retraction; tilting: anterior-posterior tilting; Abd-adduction = abduction-adduction; in-external rotation = internal-external rotation.

**Table 6:** Recommendations for parameter selection based on agreement and reliability results.

The analytical external rotation task is an appropriate task to assess ST pro-retraction and GH in-external rotation. However, thorough task explanation is essential.
The combing hair task is an appropriate task in the assessment of ST med-lateral rotation, GH flexion-extension, and elbow flexion-extension. This task is not appropriate for assessing ST pro-retraction and GH in-external rotation.
The grasping the seatbelt task is a suitable task for GH ab-adduction, GH in-external rotation, and elbow flexion-extension assessment. In contrast, grasping the seatbelt is not an option when ST pro-retraction, ST med-lateral rotation, or GH flexion-extension is assessed.
Placing a cup on an overhead shelf task is in general an appropriate task for all ST and GH joint rotations assessment. Only for elbow flexion-extension, this task does not suit.

ST = scapulothoracic; GH = glenohumeral.

## Supplementary Materials

The following are available online at https://www.mdpi.com/1424-8220/20/3/876/s1. Bland–Altman plots for intra-operator and inter-operator agreement assessment.
