# Supplementary material for: Assessment of Scapulothoracic, Glenohumeral, and Elbow Motion in Adhesive Capsulitis by Means of Inertial Sensor Technology: A Within-Session, Intra-Operator and Inter-Operator Reliability and Agreement Study
Source: Sensors (Basel). 2020 Feb 6;20(3):876. doi: 10.3390/s20030876 (PMC7038682; doi:10.3390/s20030876)
Supplement: Supplementary file 1 [file sensors-20-00876-s001.pdf]

Appendix A

<u>Analytical glenohumeral external rotation - Scapulothoracic – Protraction/Retraction</u>

Intra-operator

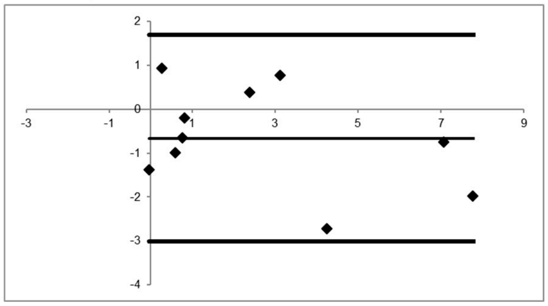

Inter-operator

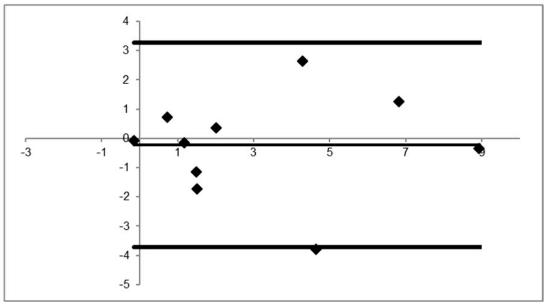

<u>Combing hair - Scapulothoracic – lateral/medial rotation</u>

Intra-operator

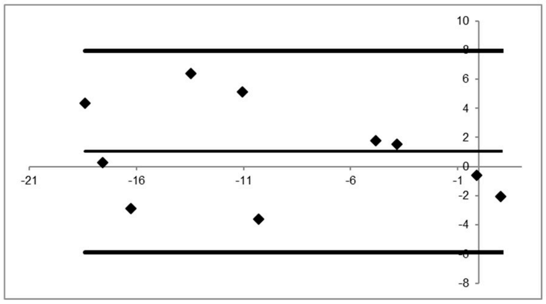

Inter-operator

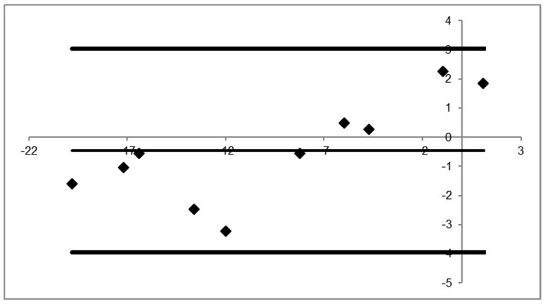

<u>Combing hair - Scapulothoracic – Protraction/Retraction</u>

Intra-operator

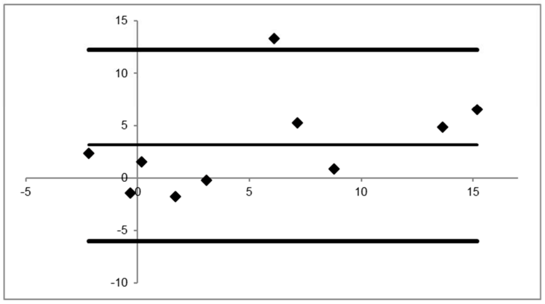

Inter-operator

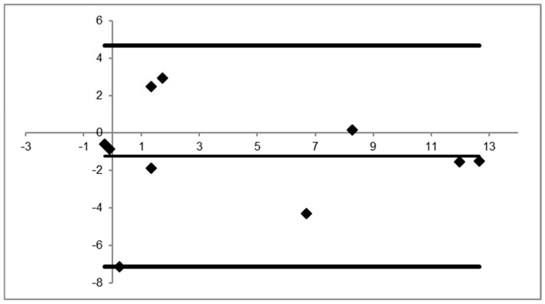

<u>Combing hair - Scapulothoracic – Posterior/anterior tilt</u>

Intra-operator

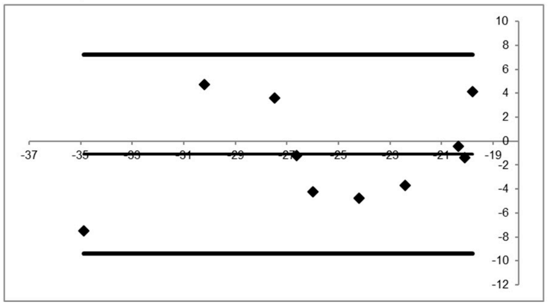

Inter-operator

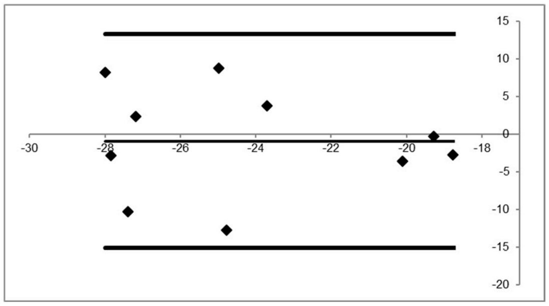

◆ Difference

Mean Difference

Mean Diff. ± 2SD

Appendix A

Grabbing a seatbelt - Scapulothoracic – lateral/medial rotation

Intra-operator

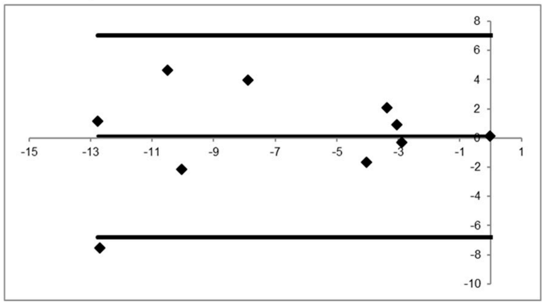

Inter-operator

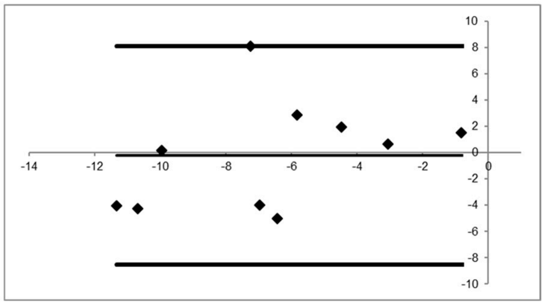

Grabbing a seatbelt - Scapulothoracic – Protraction/Retraction

Intra-operator

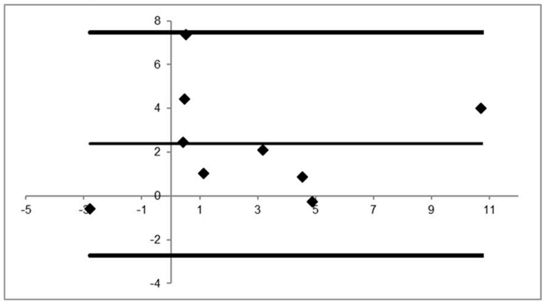

Inter-operator

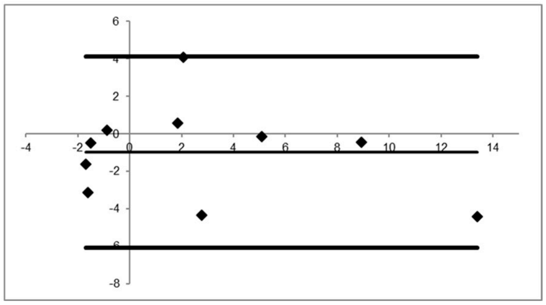

Grabbing a seatbelt - Scapulothoracic – Posterior/anterior tilt

Intra-operator

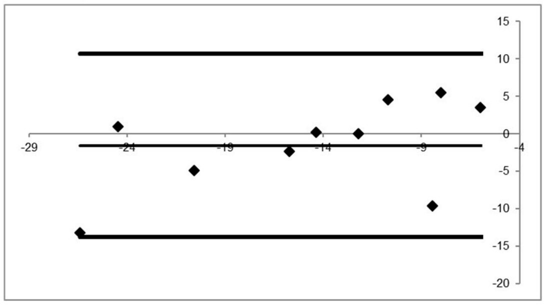

Inter-operator

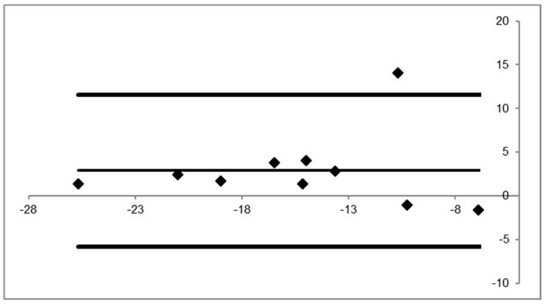

Placing a cup on an overhead shelf - Scapulothoracic – lateral/medial rotation

Intra-operator

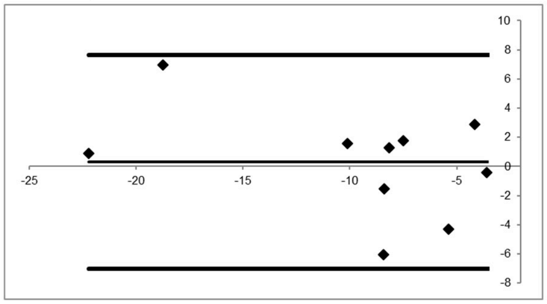

Inter-operator

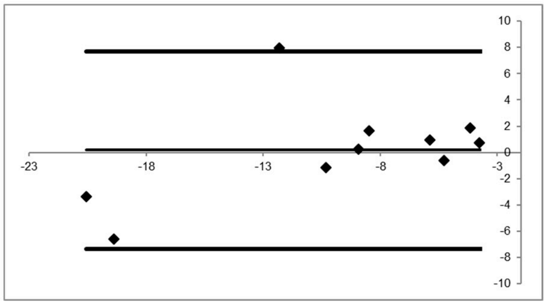

Difference
Mean Difference
Mean Diff. ± 2SD

Appendix A

Placing a cup on an overhead shelf - Scapulothoracic – Protraction/Retraction

Intra-operator

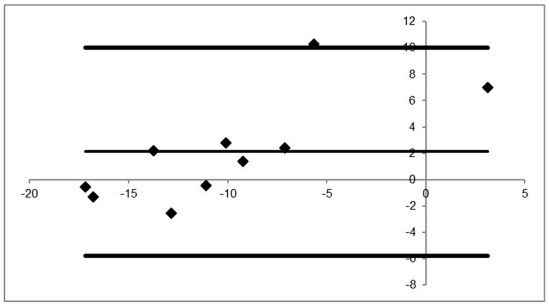

Inter-operator

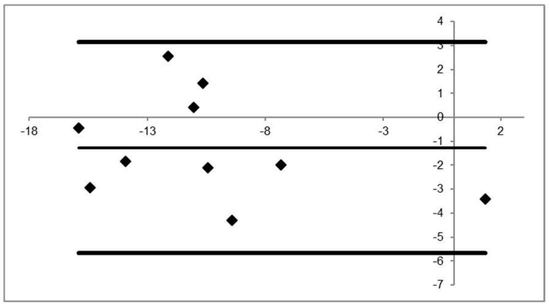

Placing a cup on an overhead shelf - Scapulothoracic – posterior/anterior tilt

Intra-operator

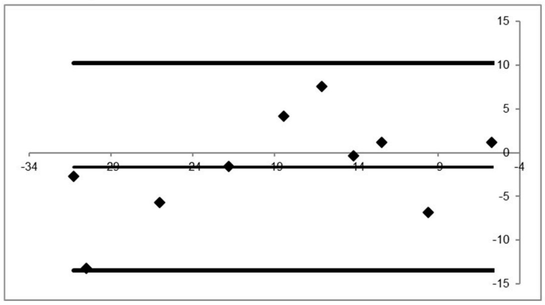

Inter-operator

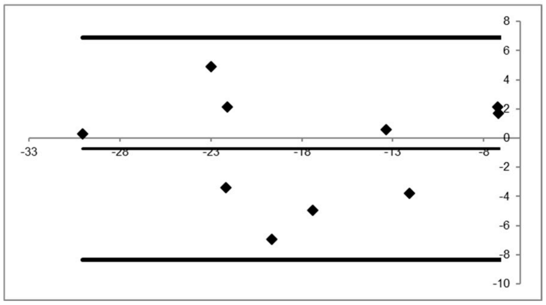

Analytical glenohumeral external rotation - Glenohumeral – internal/external rotation

Intra-operator

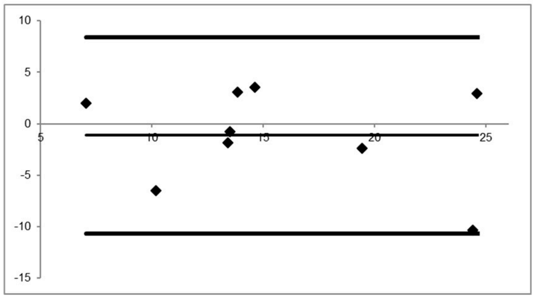

Inter-operator

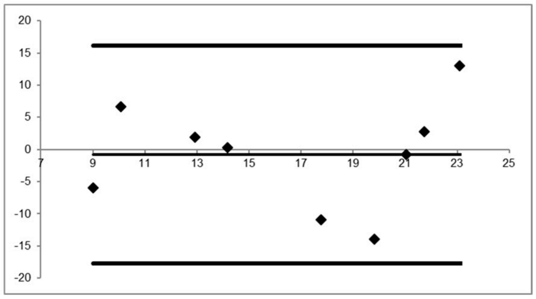

Combing hair - Glenohumeral – Abduction/adduction

Intra-operator

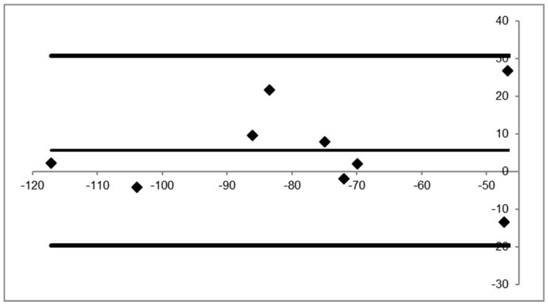

Inter-operator

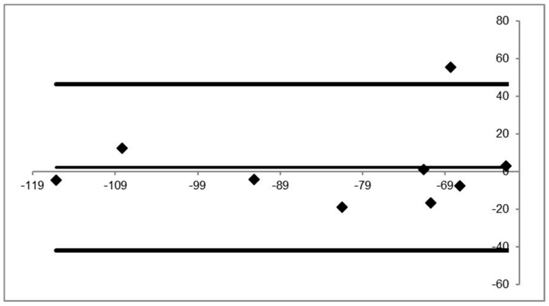

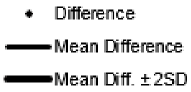

Appendix A

<u>Combing hair - Glenohumeral – Internal/External rotation</u>

Intra-operator

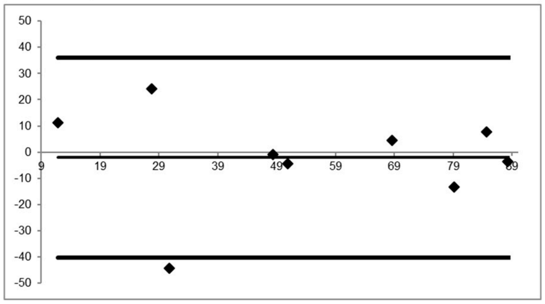

Inter-operator

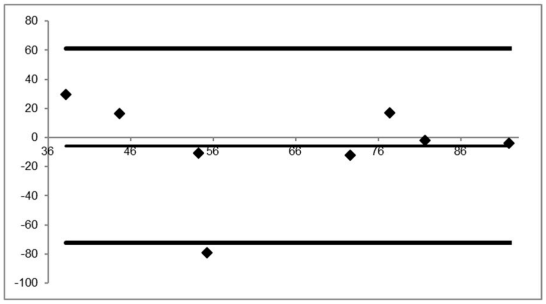

<u>Combing hair - Glenohumeral – Flexion/extension</u>

Intra-operator

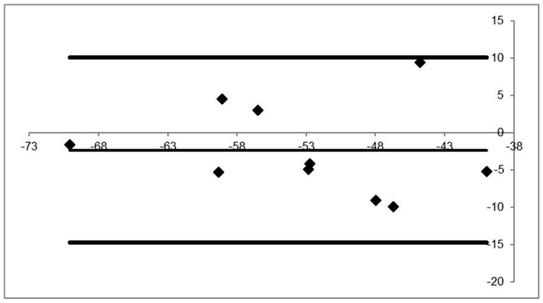

Inter-operator

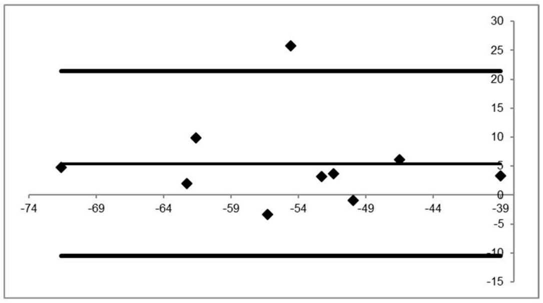

<u>Grabbing a seatbelt - Glenohumeral – Abduction/adduction</u>

Intra-operator

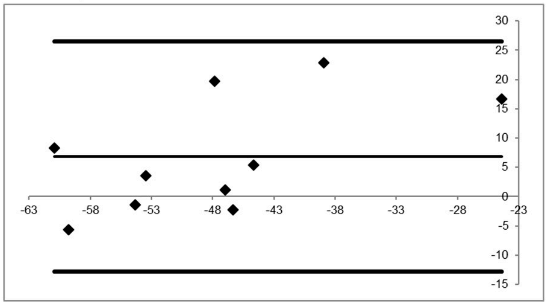

Inter-operator

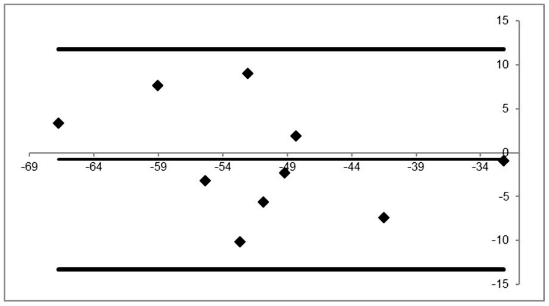

<u>Grabbing a seatbelt - Glenohumeral – internal/external rotation</u>

Intra-operator

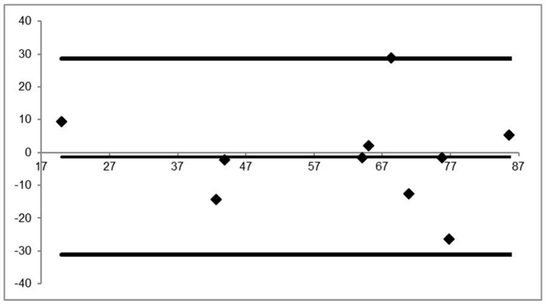

Inter-operator

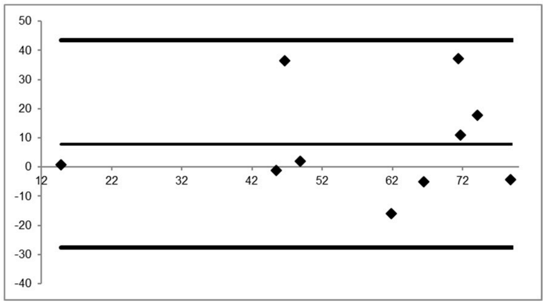

◆ Difference

Mean Difference

Mean Diff. ± 2SD

Appendix A

Grabbing a seatbelt - Glenohumeral – Flexion-extension

Intra-operator

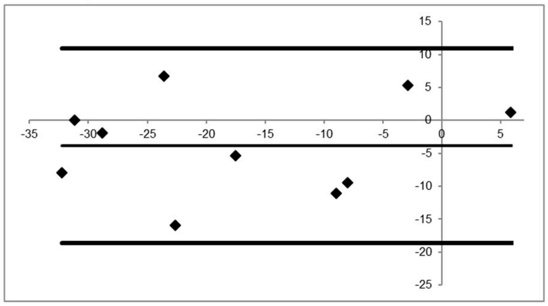

Inter-operator

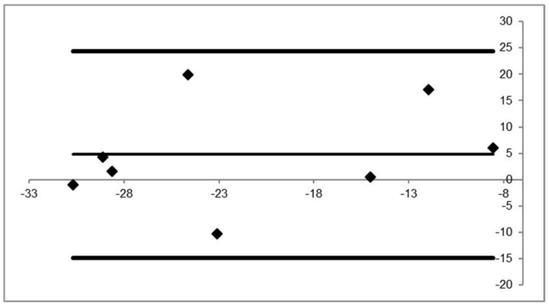

Placing a cup on an overhead shelf - Glenohumeral – Abduction/adduction

Intra-operator

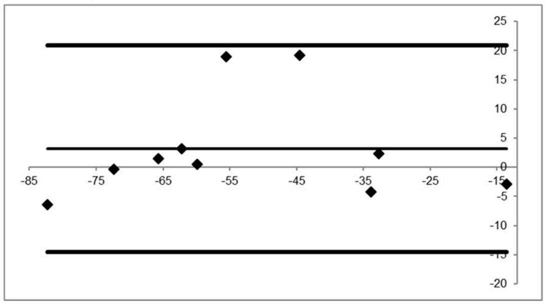

Inter-operator

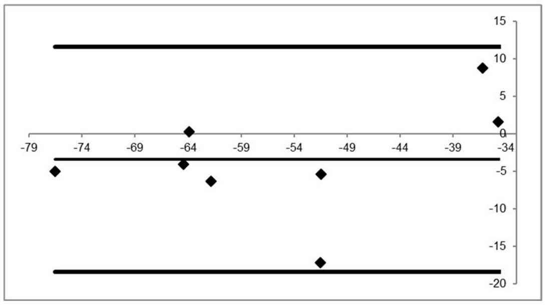

Placing a cup on an overhead shelf - Glenohumeral – internal/external rotation

Intra-operator

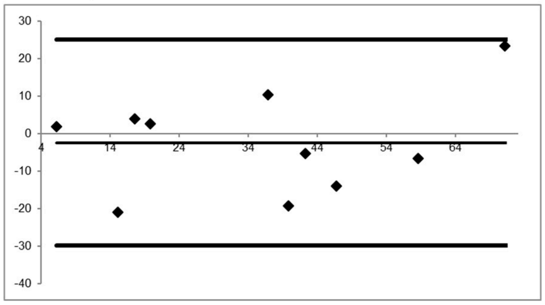

Inter-operator

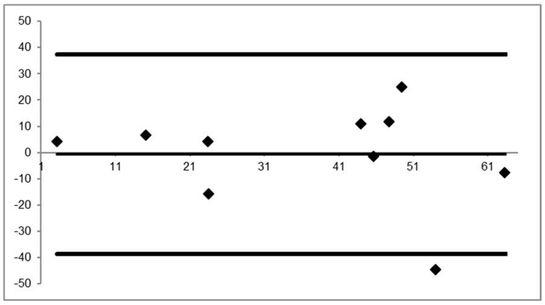

Placing a cup on an overhead shelf – Glenohumeral - Flexion-extension

Intra-operator

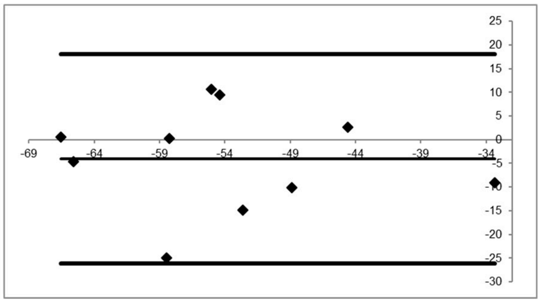

Inter-operator

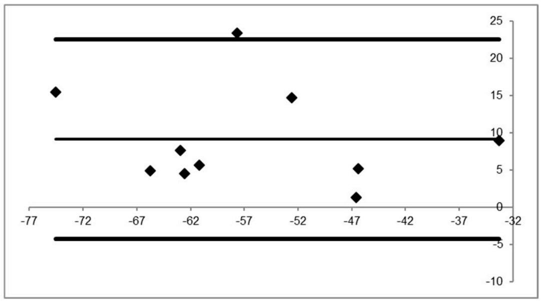

Difference

Mean Difference

Mean Diff. ±2SD

Appendix A

<u>Combing hair - Elbow – Flexion-extension</u>

Intra-operator

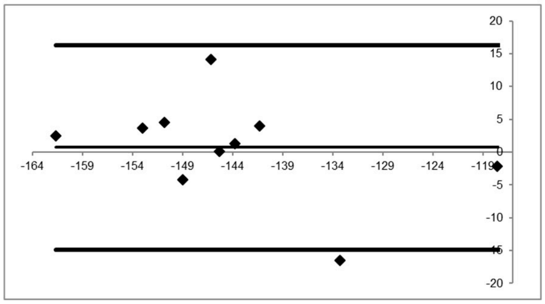

Inter-operator

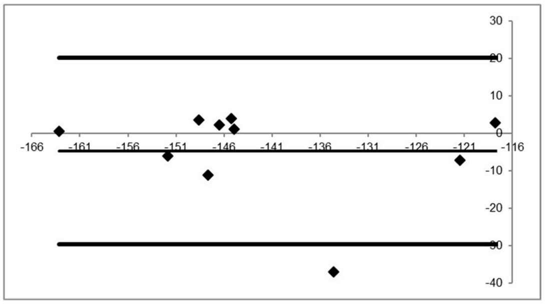

<u>Grabbing a seatbelt - Elbow – Flexion-extension</u>

Intra-operator

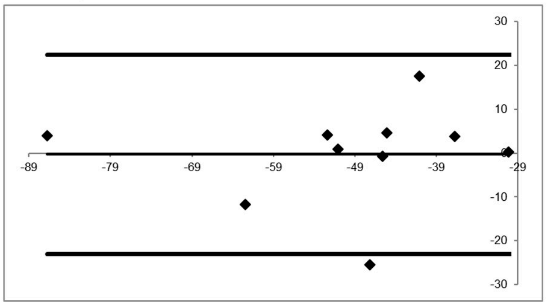

Inter-operator

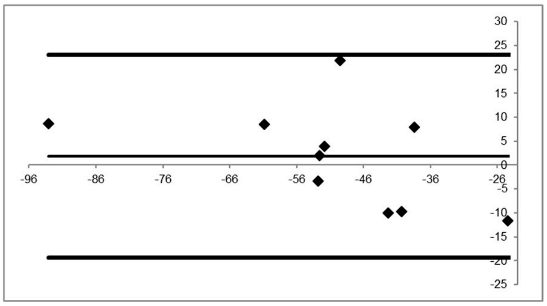

<u>Placing a cup on an overhead shelf – Elbow - Flexion-extension</u>

Intra-operator

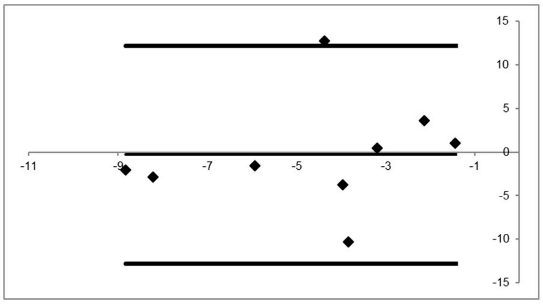

Inter-operator

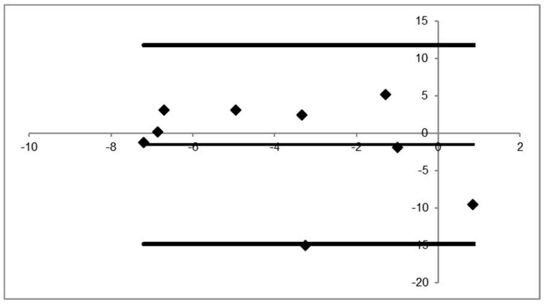

◆ Difference

— Mean Difference

— Mean Diff. ± 2SD
